# Number of functional teeth more strongly predicts all‐cause mortality than number of present teeth in Japanese older adults

**DOI:** 10.1111/ggi.13911

**Published:** 2020-03-29

**Authors:** Kenji Maekawa, Tomoko Ikeuchi, Shoji Shinkai, Hirohiko Hirano, Masahiro Ryu, Katsushi Tamaki, Hirofumi Yatani, Takuo Kuboki, Kusatsu ISLE Study Working Group Collaborators, Aya Kimura‐Ono, Takeshi Kikutani, Takashi Suganuma, Yasunori Ayukawa, Tomoya Gonda, Toru Ogawa, Masanori Fujisawa, Shoichi Ishigaki, Yutaka Watanabe, Akihiko Kitamura, Yu Taniguchi, Yoshinori Fujiwara, Ayako Edahiro, Yuki Ohara, Junichi Furuya, Junko Nakajima, Kento Umeki, Kentaro Igarashi, Yasuhiro Horibe, Yoshihiro Kugimiya, Yasuhiko Kawai, Hideo Matsumura, Tetsuo Ichikawa, Shuji Ohkawa

**Affiliations:** ^1^ Research Planning and Promotion Committee Japan Prosthodontic Society Tokyo Japan; ^2^ Okayama University Graduate School of Medicine, Dentistry, and Pharmaceutical Sciences Okayama Japan; ^3^ Tokyo Metropolitan Institute of Gerontology Tokyo Japan; ^4^ Tokyo Dental College Tokyo Japan; ^5^ Kanagawa Dental University Graduate School Yokosuka Japan; ^6^ Osaka University Graduate School of Dentistry Osaka Japan; ^7^ Okayama University Hospital Okayama Japan; ^8^ The Nippon Dental University Tokyo Japan; ^9^ Showa University School of Dentistry Tokyo Japan; ^10^ Kyushu University Faculty of Dental Science Fukuoka Japan; ^11^ Tohoku University Graduate School of Dentistry Sendai Japan; ^12^ Meikai University School of Dentistry Sakado Japan; ^13^ Hokkaido University Faculty of Dental Medicine Sapporo Japan; ^14^ National Institute for Environmental Studies Ibaraki Japan; ^15^ Tokyo Medical and Dental University Tokyo Japan; ^16^ Nihon University School of Dentistry at Matsudo Chiba Japan; ^17^ Nihon University School of Dentistry Tokyo Japan; ^18^ Tokushima University Graduate School Institute of Biomedical Sciences Tokushima Japan

**Keywords:** functional teeth, mortality, present teeth, risk factor

## Abstract

**Aim:**

Previous studies on the association between intraoral conditions and mortality in community‐dwelling older individuals reported that fewer present teeth (PT) are significant risk factors for mortality. However, how the number of PT relative to the number of functional teeth (FT), including both present and rehabilitated teeth, influences mortality has not been investigated fully. This study examined the impact of the number of FT on mortality among community‐dwelling Japanese older adults.

**Methods:**

This study was a retrospective, observational and population‐based follow‐up study, which examined 1188 older individuals who participated in an annual geriatric health examination from 2009 to 2015. The average follow‐up period was 1697.0 ± 774.5 days. The primary outcome was all‐cause mortality at follow‐up. The numbers of PT and FT of each participant were counted during an oral examination. In addition, demographics, clinical variables, blood nutrient markers, physical functions and perceived masticatory function were measured.

**Results:**

Kaplan–Meier analysis, followed by a log‐rank test, revealed that fewer PT (*P* < 0.001) and FT (*P* = 0.002) were significantly associated with a reduced survival rate. Cox's proportional hazard analysis indicated that the number of FT, but not the number of PT, was a significant independent mortality risk factor after adjusting for demographics, clinical variables, nutrient markers and physical functioning (*P* = 0.036, hazard ratio: 2.089).

**Conclusions:**

Current results suggest that the number of FT more strongly predicts all‐cause mortality than the number of PT among community‐dwelling older adults. Further studies are necessary to consider the confounding of socioeconomic status and disability status. **Geriatr Gerontol Int 2020; ••: ••–••**.

## Introduction

An increasing number of studies on the association between tooth loss and mortality among community‐dwelling older individuals have reported that fewer present teeth (PT) are a significant risk factor for mortality.^1^, ^2^ Fewer teeth are linked with nutritional disturbance, which can be improved by increasing the number of functional teeth (FT) by installing rehabilitated teeth.^3^ Thus, prosthodontic treatment could potentially reduce mortality risk, even if the number of PT decreases. However, this prediction has not been scientifically verified. A study with a sufficient sample size of community‐dwelling older adults reported that wearing complete dentures affected mortality risk among edentulous individuals. Results demonstrated that complete dentures decreased the mortality hazard ratio (HR) by 42% after adjusting for potential covariates among community‐dwelling older adults.^4^ Furthermore, Yoshida *et al*. reported the effects of three occlusal contact patterns between the maxilla and mandible, and the effects of wearing dentures on mortality risk, among fully and partially edentulous community‐dwelling older adults. Results demonstrated that individuals without dentures, and any natural tooth contact between the maxilla and mandible was absent, had a 1.52‐fold increase in mortality risk after adjusting for age and gender.^5^

These previous studies examined whether prosthodontic treatment for missing teeth is effective for elongating longevity among community‐dwelling older adults. However, no previous study has examined differences in how the number of PT relative to the number of FT influences mortality. This lack of research may be due to difficulties in counting the net number of PT with no pontic teeth, fixed bridge restorations or implant‐supported superstructures.

Here, we examined the relationship between the number of FT and PT, and mortality while adjusting for potential covariates, including demographics, history of systemic disease, nutritional status, and psychosocial, physical and masticatory functioning among community‐dwelling older adults in Japan.

## Methods

### *Participants*

This study was conducted as a retrospective, observational and population‐based follow‐up study and the participants were selected from community‐dwelling older individuals aged ≥65 years who participated in a comprehensive geriatric health examination conducted annually in the town of Kusatsu (total population in 2015, *n* = 6518; 37.1% aged ≥65 years) in Gunma prefecture, Japan, between 2009 and 2015. Most participants underwent an annual examination multiple times during this period. Thus, data from the first examination of each participant served as a baseline. In total, 1240 participants (539 men and 701 women; mean ± SD age = 76.6 ± 7.1 years) were included in the analyses.

All participants provided written informed consent under conditions approved by the ethics committee of the Tokyo Metropolitan Institute of Gerontology (Issue no. 3 in 2008).

### *Examination measures*

The variables analyzed in this study were sex, age, history of chronic diseases, hemoglobin, albumin, total cholesterol, Geriatric Depression Scale (GDS) short‐version,^6^ Mini–Mental State Examination (MMSE),^7^ gait speed, hand‐grip strength and self‐perceived chewing ability. Chronic diseases included clinically relevant medical conditions: hypertension, hyperlipidemia, cerebral vascular disease, heart disease, diabetes mellitus and cancer. For each condition, participants were asked if they had received a diagnosis from a physician (yes or no).

We measured 15‐item GDS scores, ranging from 0 to 15, using a self‐report questionnaire. Depressive levels for each participant were divided into two categories (<6 and ≥6) using a GDS score of 6 as the cut‐off.^6^ The MMSE is widely used as a screening test for dementia and general cognitive functioning.^7^ MMSE scores were recorded during face‐to‐face interviews with participants. The MMSE consists of 11 items, and scores representing individual cognitive function range from 0 to 30. Based on a cut‐off level >24, participants were divided into two groups (>24 and ≤24).^8^

After the medical interview, body height and weight were measured, and blood tests were performed. Body mass index (BMI) was then manually calculated using the formula: body weight (kg)/height (m).^2^ Participants were categorized into three groups (<20.0, 20.0–24.9, ≥25.0) then submitted for subsequent analyses.^9^ Each blood nutrient marker was categorized into two groups using the following cut‐off values (hemoglobin, male: 13.0 g/dL [<13.0 and ≥13.0 g/dL], female: 12.0 g/dL [<12.0 and ≥12.0 g/dL];^10^ albumin: 4.0 g/dL [<4.0 and ≥4.0 g/dL];^11^ total cholesterol, male: 160 mg/dL [<160 and ≥160 mg/dL], female: 180 mg/dL [<180 and ≥180 mg/dL]). Lower cholesterol levels in older adults are reported to be associated with increased mortality risk.^12^ Thus, we referred to the National Health and Nutrition Survey Japan, indicating that approximately 10% of older men and women fell under 160 and 180 mg/dL, respectively, and used these values as the cut‐off for total cholesterol.

Gait speed and hand‐grip strength data were collected as physical performance measures. Participants walked along a straight, 11‐m walkway on a flat floor at their usual speed. Usual gait speed was measured over the 5 m distance between markers placed at 3 and 8 m from the starting point of the walkway.^13^ Participants were categorized into two groups (<1.0 and ≥1.0 m/s) using a cut‐off speed of 1.0 m/s.^14^ Grip strength of participants’ dominant hand was measured (kilogram force, i.e., kgf), and the highest value of two trials was used to categorize into two levels based on established cut‐off levels (male: 26.0 kgf; female: 18.0 kgf).^15^

Self‐perceived chewing ability was measured with a question assessing the ability to chew foods;^16^ “Do you have difficulties in chewing food? If you are using dentures, please answer the question under the circumstances of denture use.” Participants selected from four responses: (i) no difficulties; (ii) some difficulties, but can eat most foods; (iii) difficulties, and can eat limited foods, and (iv) cannot chew much. As only three participants answered (iv), those who answered (iii) or (iv) were combined into one group.

### *Oral examination*

The oral examination, including counting the number of PT and FT, which contained natural remaining teeth and prosthetically restored missing teeth,^17^ was carried out by well‐trained and calibrated, professionally registered dentists in a blinded fashion to the other medical predictors. Third molars were also counted; hence, the numbers of PT and FT ranged from 0 to 32. Stump teeth and severely decayed teeth not used for mastication were excluded from this definition.

PT in this study did not count dummy teeth on fixed partial dentures or implant‐supported artificial teeth, while FT included the functioning PT plus the number of artificial teeth on removable dentures being worn during the oral examination, dummies on fixed partial dentures and implant‐supported artificial teeth.^18^ Based on the number of PT, participants were assigned to three groups,i.e.,^19^ 0–9, 10–19 and ≥20 teeth. A previous study has suggested that having ≥20 teeth is satisfactory for any individual's chewing ability,^20^ so participants were also assigned to two groups based on the number of FT, i.e., ≤19 and ≥20.

### *Mortality*

The primary outcome of interest in this study was all‐cause mortality at follow‐up. Local registries, maintained by every municipality and reported monthly, were examined to verify deaths from any cause. Participants were followed until death or the end of follow‐up (February 2016) and divided into mortality or survival groups.

### *Statistical analyses*

Chi‐squared tests and *t*‐tests were utilized to compare baseline predictors between the two groups (categorized by the number of FT). Fisher's exact test instead of chi‐squared test was used when the smallest of the expected numbers was <5. Survival rates were calculated using a Kaplan–Meier analysis. The log‐rank test was used to compare the survival curves between two groups. Finally, Cox (or proportional hazards) regression models were used to calculate the HR for mortality. Six analytical models were employed: model 0 (crude, unadjusted model), only the numbers of PT and FT were submitted as predictors; model 1, adjusted for age and sex; model 2, adjusted for all the variables in model 1 plus clinically relevant variables, such as chronic diseases, depression and cognitive function; model 3, adjusted for all the variables in model 2 plus BMI and blood nutrition; model 4, adjusted for all the variables in model 3 plus physical performance measures; and model 5, adjusted for all the variables in model 4 plus self‐perceived chewing ability. Models 3, 4 and 5 were set to determine whether nutritional status, physical performance and chewing ability would mediate at least in part the association of the number of teeth and mortality. The significance level was set at *P* < 0.05. All statistical analyses were performed using IBM SPSS Statistics (Version 25.0; IBM Corp., Armonk, NY, USA).

## Results

### *Study flow*

**Figure 1:**
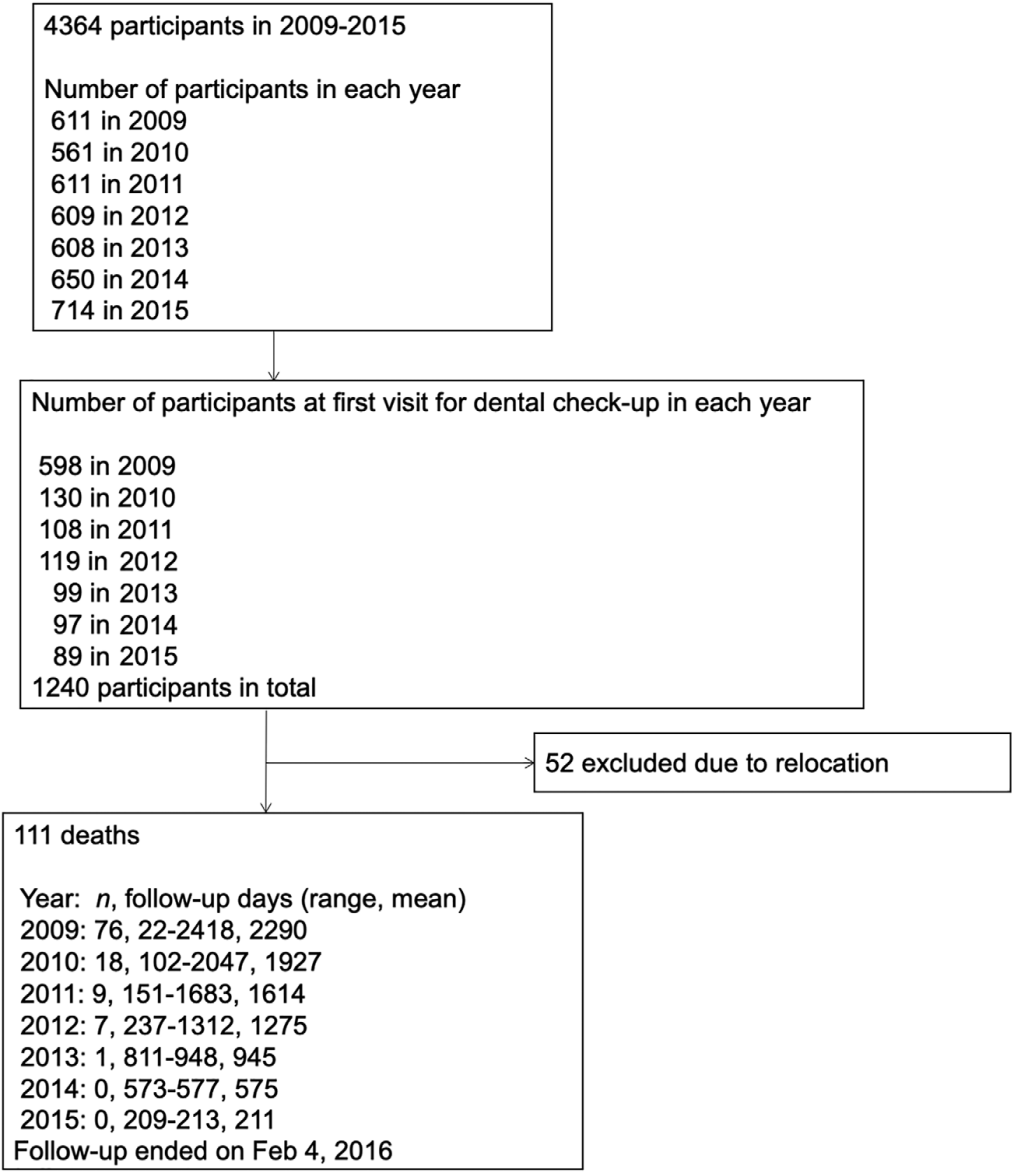
Participant flowchart.

In total, 4364 individuals participated in the annual health examination between 2009 and 2015. At baseline, 1240 participants were recruited for the health examination. During the follow‐up period, 52 participants dropped out because of relocation and were excluded from the sample. Thus, in total, 1188 participants were examined for further analysis (Fig. 1).

### *Participant characteristics*

Table 1 shows baseline properties compared between groups categorized by the numbers of the FT (0–19 and ≥20). Significant proportion rate differences were observed in mortality, the number of PT, history of hyperlipidemia, cognitive function and self‐perceived chewing ability as main results between two groups. In addition, mean age was significantly different between the groups.

**Table 1:** Comparisons of baseline properties between the groups categorized by the number of functional teeth

Variables	Functional teeth		*P*‐value
	0–19	≥20	
Mortality, *n* (%)	13 (19.4)	98 (8.7)	**0.004** †
Numbers of present teeth			**<0.001** †
0–9, *n* (%)	39 (58.2)	427 (38.0)	
10–19, *n* (%)	28 (41.8)	216 (19.3)	
≥20, *n* (%)	0 0	477 (42.6)	
Age (years), mean ± SD	73.4 (6.5)	72.1 (6.8)	0.697‡
Gender (men), *n* (%)	39 (58.2)	474 (39.9)	**0.011** †
History of chronic disease			
HT (hypertension), *n* (%)	20 (29.8)	459 (40.9)	0.069†
Hyperlipidemia	7 (10.4)	247 (22.0)	**0.024** †
Stroke, *n* (%)	7 (10.4)	55 (4.9)	0.055§
Heart disease, *n* (%)	7 (10.4)	108 (9.6)	0.832†
Diabetes mellitus, *n* (%)	8 (11.9)	118 (10.5)	0.692†
Cancer, *n* (%)	5 (7.5)	93 (8.3)	0.814†
Depressive mood (GDS score ≥6), *n* (%)	16 (23.9)	208 (18.6)	0.165†
Cognitive function (MMSE score <24), *n* (%)	18 (26.9)	174 (15.5)	**0.008** †
Body mass index			0.1†
<20.0, *n* (%)	14 (20.9)	169 (15.1)	
20.0–24.9, *n* (%)	32 (47.8)	683 (60.1)	
≥25.0, *n* (%)	21 (31.3)	269 (24.0)	
Blood nutritional parameters			
Albumin (<4.0), *n* (%)	9 (13.4)	151 (13.5)	0.953†
Hemoglobin			0.118†
Men (<13.0), *n* (%)	4 (10.3)	43 (9.1)	
Women (<12.0), *n* (%)	6 (21.4)	58 (8.1)	
Total cholesterol			0.337†
Men (<160), *n* (%)	8 (20.5)	61 (12.9)	
Women (<180), *n* (%)	5 (17.9)	104 (14.6)	
Gait speed (m/s)			0.152†
<1.0, *n* (%)	12 (17.9)	147 (13.1)	
Grip strength			0.513†
Men (≥26.0), *n* (%)	4 (10.3)	35 (7.4)	
Women (≥18.0), *n* (%)	9 (32.1)	163 (22.8)	
Self‐perceived chewing ability			**<0.001** †
Cannot chew much, *n* (%)	2 (3.0)	1 (0.1)	
Can eat only limited food, *n* (%)	11 (16.4)	37 (3.3)	
Can eat most foods, *n* (%)	33 (49.3)	326 (29.1)	
No difficulties, *n* (%)	19 (28.4)	739 (65.9)	

GDS, Geriatric Depression Scale short version (15 items); MMSE, Mini–Mental State Examination (30‐point questionnaire); *n*, number of individuals; SD, standard deviation.

Number of functional teeth: 0–19 (*n* = 67), ≥20 (*n* = 1121).

*P*‐values that were statistically significant are indicated as bold letters with under bars.

† Chi‐squared test.

‡ *t*‐test.

§ Fisher's exact test.

### *Kaplan–Meier survival estimates*

**Figure 2:**
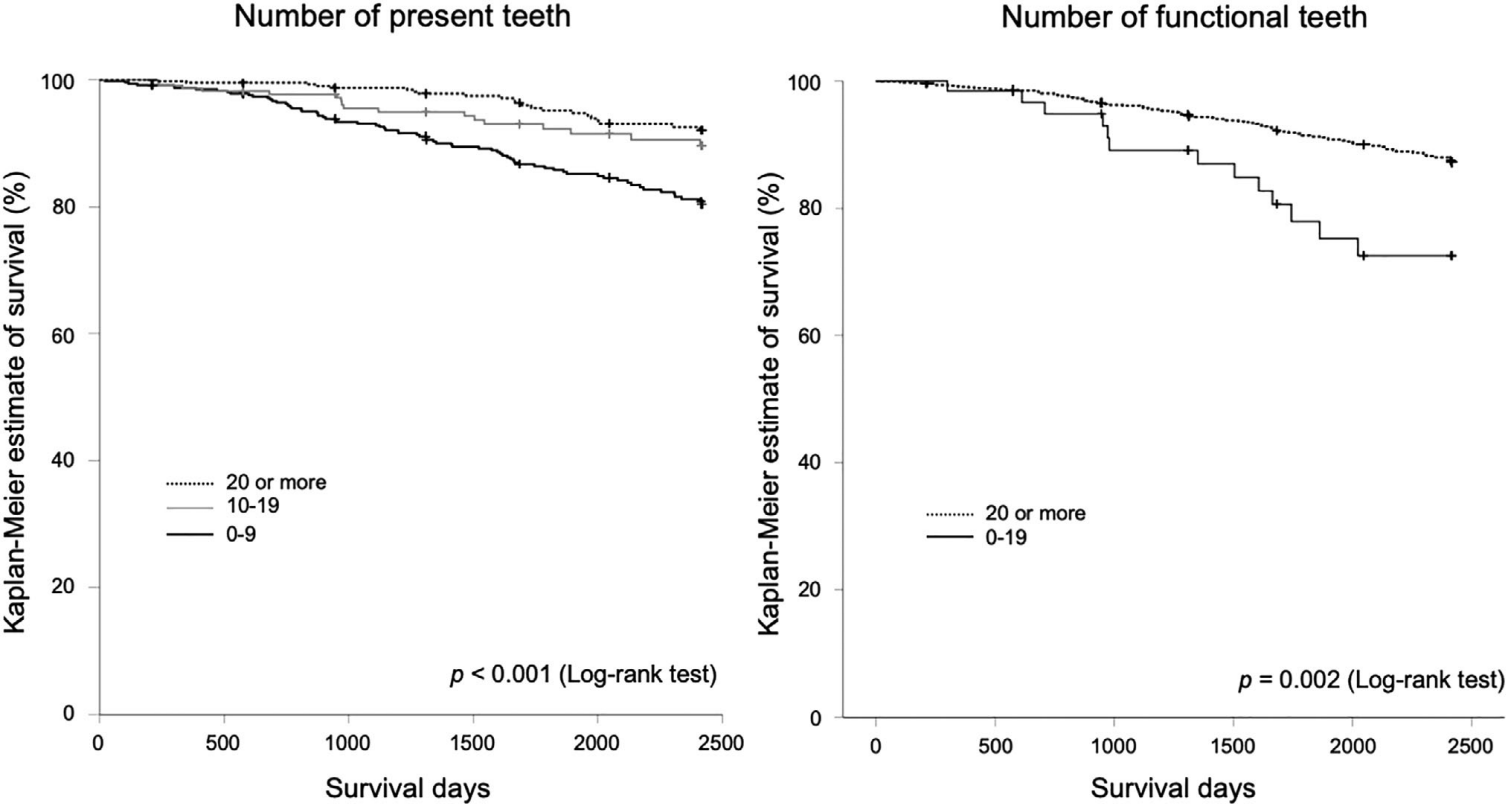
Kaplan–Meier estimate of survival curves of the participants categorized by number of present teeth (left) and number of functional teeth (right). Significant differences were determined using log‐rank tests (number of present teeth: *P* < 0.001, number of functional teeth: *P* = 0.002).

In total, 118 participants died during the follow‐up period, and the average follow‐up duration was 1697.0 ± 774.5 days. Figure 2 shows the Kaplan–Meier estimate of participant survival. The left side shows the Kaplan–Meier estimate categorized by the number of PT. The right side depicts the Kaplan–Meier estimate categorized by the number of FT. This analysis indicated that the Kaplan–Meier estimates of participants with more PT and FT were significantly higher than those participants with fewer teeth were (log‐rank test, PT: *P* < 0.001; and FT: *P* = 0.002).

### *Mortality risk factors*

Based on the finding of significant associations between the numbers of PT and FT and mortality, Cox proportional hazard analyses were performed to assess whether these two predictors could be used as real predictor variables of mortality. In model 0, fewer PT (0–9: *P* < 0.0001, HR: 2.645) and FT (*P* = 0.019, HR: 2.026) were significant risk factors for mortality (Table 2). However, fewer PT were not a significant risk factor in all subsequent analytical models (1–5) that adjusted for other variables. In contrast, fewer FT were a significant risk factor in models 1, 2 and 4 and were linked to higher HRs (Table 2). Detailed statistical results for each model, which found significant risk factors for mortality, are shown below.

- *Model 1*: fewer FT (*P* = 0.031, HR: 1.923), older age (*P* < 0.0001, HR: 1.121) and being male (*P* < 0.0001, HR: 2.478) were significant risk factors.
- *Model 2*: similar to model 1, fewer FT (*P* = 0.042, HR: 1.946), older age (*P* < 0.0001, HR: 1.114) and being male (*P* < 0.0001, HR: 2.669) were significant risk factors.
- *Model 3*: older age (*P* < 0.0001, HR: 1.096), being male (*P* < 0.0001, HR: 2.755), lower blood albumin (*P* = 0.002, HR: 2.014) and lower total blood cholesterol (*P* = 0.049, HR: 1.584) were significant risk factors. The number of FT was not a significant risk factor (*P* = 0.058, HR: 1.888).
- *Model 4*: fewer FT (*P* = 0.036, HR: 2.089), older age (*P* < 0.0001, HR: 1.080), being male (*P* < 0.0001, HR: 3.364) and lower total blood hemoglobin (*P* = 0.002, HR: 2.129) were significant risk factors.
- *Model 5*: older age (*P* < 0.0001, HR: 1.078), being male (*P* < 0.0001, HR: 3.691), lower blood albumin (*P* = 0.003, HR: 2.073), slower gait speed (*P* = 0.047, HR: 1.655) and lower self‐perceived chewing ability (*P* = 0.043, HR: 2.170) were significant risk factors. The number of FT was not a significant risk factor (*P* = 0.302, HR: 1.504).

**Table 2:** Hazard ratios and 95% confidence intervals of the numbers of present and functional teeth submitted as predictors for each Cox proportional hazard model

	Model 0	Model 1	Model 2	Model 3	Model 4	Model 5
Present teeth						
≥20	1.0 [Reference]	1.0 [Reference]	1.0 [Reference]	1.0 [Reference]	1.0 [Reference]	1.0 [Reference]
10–19	1.318 (0.693–2.504)	1.071 (0.557–2.056)	1.069 (0.543–2.102)	1.210 (0.602–2.431)	1.274 (0.622–2.608)	1.418 (0.693–2.901)
0–9	**2.645 (1.629–4.294)**	1.637 (0.979–2.737)	1.532 (0.896–2.620)	1.694 (0.982–2.925)	1.656 (0.940–2.918)	1.612 (0.899–2.890)
Functional teeth						
≥20	1.0 [Reference]	1.0 [Reference]	1.0 [Reference]	1.0 [Reference]	1.0 [Reference]	1.0 [Reference]
0–19	**2.026 (1.123–3.655)**	**1.923 (1.060–3.489)**	**1.946 (1.026–3.692)**	1.888 (0.979–3.642)	**2.089 (1.051–4.153)**	1.504 (0.693–3.263)

Model 0 shows the crude, unadjusted results. Model 1 is adjusted for age and sex. Model 2 is adjusted for all the variables in model 1 plus clinically relevant variables (history of hypertension, hyperlipidemia, stroke, heart disease, diabetes, cancer, depression and cognitive function). Model 3 is adjusted for all the variables in model 2 plus body mass index and nutrition markers (albumin, hemoglobin, and cholesterol). Model 4 is adjusted for all the variables in model 3 plus physical function (usual gait speed and hand grip strength). Model 5 is adjusted for all the variables in model 4 plus subjective masticatory function.

Hazard ratios that were statistically significant (*P* < 0.05) are indicated as bold letters and with under bar.

## Discussion

The current study investigated the relationship between intraoral status and mortality among community‐dwelling older individuals using the number of FT as a predictor in conjunction with the number of PT. A Kaplan–Meier analysis indicated that survival estimates among those groups with fewer PT and/or FT was significantly lower than among those with more teeth. These findings are consistent with previous studies on number of PT.^1^, ^2^ Given that the relationship between the number of FT and mortality has not been examined, to the best of our knowledge, the current study provides the first evidence that fewer FT is related to mortality.

Results of the Cox proportional hazard models, adjusting for multiple variables, revealed that being older, being male, and having lower nutrient markers and lower physical functioning were significant risk factors for mortality, consistent with previous findings.^21^ This may suggest that results from the current sample have high external validity. The results also indicated that having fewer FT significantly increased mortality risk. Meanwhile, fewer PT, which is commonly recognized as a risk factor, did not represent a significant risk factor after adjusting for multiple factors, including the number of FT. However, a decrease in the number of FT was not a significant risk factor after adjusting for self‐perceived chewing ability. Instead, lower self‐perceived chewing ability was confirmed as a significant risk factor. These findings suggest that both the number of FT and self‐perceived chewing ability may have moderating effects on mortality. Self‐perceived chewing difficulties could represent a powerful and direct factor inducing actual nutritional problems.

Based on the current findings, the following issues must be considered. First, a decreased number of PT could increase mortality risk through the induction of masticatory and subsequent nutritional intake difficulties, possibly exacerbating general conditions, accelerating frailty, loss of independence and disease onset. However, while fewer FT were a significant risk factor in several statistical models in the present study, fewer PT were no longer a significant predictor after controlling for multiple variables. This suggests that prosthodontic rehabilitation could reduce mortality risk, even after tooth loss.

Second, the current results provide new insight regarding potential causal pathways between tooth loss and mortality. Two pathways have been previously considered for the causal relationship between the two factors.^22^ First, nutritional problems may arise because of masticatory dysfunction induced by tooth loss, resulting in the onset of systemic diseases, leading to death.^23^ Another potential pathway or cascade is via the involvement of intraoral chronic inflammation (periodontitis). This is a serious cause of not only tooth loss but also the spread of infection to the cardiovascular system^24^ and is known to aggravate other systemic diseases such as diabetes and kidney disease.^25^, ^26^ Therefore, tooth loss may be a surrogate endpoint for the deterioration of general health due to the progression of periodontitis, thus increasing mortality risk.^27^ While these two pathways have been considered in previous studies, the current findings support a proposed theoretical pathway involving nutritional failure secondary to masticatory dysfunction, with the cascade playing a more significant role in increasing mortality risk. This assumption is also supported by our Cox hazard analyses (model 3), indicating that lower blood nutrient markers were significant risk factors for mortality, even though the number of FT no longer presented as a significant risk factor in the statistical model. The number of FT was no longer a significant risk factor for mortality after controlling for self‐perceived chewing ability. This finding potentially indicates that intuitive self‐evaluated chewing ability more accurately reflects extant masticatory function and nutritional status compared with the number of teeth examined intraorally.

The current study was limited due to the lack of consideration of socioeconomic status (SES) and mortality. Because people who are more affluent tend to receive dental treatment,^28^ older people in low SES may have fewer PT and FT. In addition, people with lower SES generally show higher mortality rates than people with higher SES.^29^ Thus, the present result that higher mortality among people with fewer FT could also be explained by the confounding of socioeconomic conditions. Furthermore, the confounding of SES between teeth and mortality might be stronger for FT than PT because people with fewer FT can be the population with fewer PT, who lack access to adequate dental care. Therefore, these people could be considered as an extreme population, different from those with fewer PT but with an adequate number of FT. However, as general dental services are covered by the universal health insurance system in Japan, this influence might be smaller than in those countries without universal health coverage. In addition, disability status could be another confounder, considering that disabled older people are less likely than non‐disabled older people to obtain adequate dental treatment. Future studies are necessary to clarify these relationships. Another limitation is the absence of denture quality evaluation. Despite the fact that most participants wore dentures, wearing ill‐fitting dentures has been linked to lower quality and intake of certain nutrients.^30^ Future studies are needed to evaluate denture quality, given that dentures serve as prostheses for increasing the number of FT.

Meanwhile, differential counts of PT and FT are sometimes difficult in epidemiological settings, due to dummies and implant‐supported artificial teeth that are being mistaken for PT. In this study, prosthodontic specialists supported the intraoral examination‐based identification of dummies and artificial teeth, contributing to a greater reliability and validity in differential identification of the relationship between PT and FT and mortality. Furthermore, to the best of our knowledge, this is the first study to provide evidence that functional rehabilitation via prosthodontic treatment after tooth loss could extend longevity among community‐dwelling older adults.

In conclusion, the current results indicate that the number of FT predicts mortality among community‐dwelling older Japanese adults. This suggests that increasing the number of FT through prosthetic rehabilitation could mitigate or cancel the negative effects of fewer PT on mortality risk by improving masticatory function and nutrient status. Therefore, the causal effect of the number of FT on mortality risk may be mediated by masticatory function and nutrient status. Future studies with a larger sample size and appropriate analytical model should investigate this indirect causal path and its magnitude between the number of teeth and mortality.

## Disclosure statement

The authors declare no conflicts of interest.
